# Exploring safety systems for dispensing in community pharmacies: Focusing on how staff relate to organizational components^☆^

**DOI:** 10.1016/j.sapharm.2014.06.005

**Published:** 2015-03

**Authors:** Jasmine Harvey, Anthony J. Avery, Darren Ashcroft, Matthew Boyd, Denham L. Phipps, Nicholas Barber

**Affiliations:** a Nuffield Department of Primary Care Health Sciences, University of Oxford, Oxford, UK; b Division of Primary Care, School of Medicine, University of Nottingham, Queens Medical Centre, Nottingham, UK; c School of Pharmacy & Pharmaceutical Sciences, University of Manchester, Oxford Road, Manchester, UK; d School of Pharmacy, University of Nottingham, East Drive, University Park, Nottingham, UK; e School of Pharmacy, Department of Practice and Policy, University College London, London, UK

**Keywords:** Community pharmacy, Human factors, Patient safety, Clinical safety management

## Abstract

**Background:**

Identifying risk is an important facet of a safety practice in an organization. To identify risk, all components within a system of operation should be considered. In clinical safety practice, a team of people, technologies, procedures and protocols, management structure and environment have been identified as key components in a system of operation.

**Objectives:**

To explore risks in relation to prescription dispensing in community pharmacies by taking into account relationships between key components that relate to the dispensing process.

**Methods:**

Fifteen community pharmacies in England with varied characteristics were identified, and data were collected using non-participant observations, shadowing and interviews. Approximately 360 hours of observations and 38 interviews were conducted by the team. Observation field notes from each pharmacy were written into case studies. Overall, 52,500 words from 15 case studies and interview transcripts were analyzed using thematic and line-by-line analyses. Validation techniques included multiple data collectors co-authoring each case study for consensus, review of case studies by members of the wider team including academic and practicing community pharmacists, and patient safety experts and two presentations (internally and externally) to review and discuss findings.

**Results:**

Risks identified were related to relationships between people and other key components in dispensing. This included how different levels of staff communicated internally and externally, followed procedures, interacted with technical systems, worked with management, and engaged with the environment. In a dispensing journey, the following categories were identified which show how risks are inextricably linked through relationships between human components and other key components: 1) dispensing with divided attention; 2) dispensing under pressure; 3) dispensing in a restricted space or environment; and, 4) managing external influences.

**Conclusions:**

To identify and evaluate risks effectively, an approach that includes understanding relationships between key components in dispensing is required. Since teams of people in community pharmacies are a key dispensing component, and therefore part of the operational process, it is important to note how they relate to other components in the environment within which they operate. Pharmacies can take the opportunity to reflect on the organization of their systems and review in particular how they can improve on the four key categories identified.

## Introduction

Previous studies^1,2^ have depicted the community pharmacy as an organization where networks of people, technical and other components in the environment come together to achieve a shared objective: dispense safely to patients. To achieve safe dispensing, pharmacy managers engage in safety practices that are part of a safety system. A safety system reflects the organization's commitment to safety and is a key ingredient in employees' perceptions about the importance of safety.^3,4^ In a community pharmacy, a safety system may include a set of values about safety,^5^ employees' safety behavior,^6–8^ protocols and operational rules,^1,9^ preventative planning such as identifying and evaluating risk,^3,4,10,11^ and root cause incident analysis for organizational learning.^12,13^ In this article, ‘risk’ is used to depict ‘acts or conditions’ that influence how safety is practice and achieved.

Therefore, the networks of people and their attitudes, organizational factors affecting them and environments within which they operate are all components that should be considered when identifying and evaluating risk as part of a safety system.^11,14,15^ According to Reason's Swiss Cheese model,^16^ these components can be organized into five “layers”:

1. *Decision-makers*: the strategic decision-making that determines what staff will do and how;
2. *Line management*: the implementation of strategic decisions by directing work activities;
3. *Preconditions*: the prerequisites for successful work activity activities to be successful (for example equipment, training and procedures);
4. *Work activities*: the behaviors that are carried out;
5. *Defenses*: the safeguards put in place to protect people and equipment from hazards associated with the work activities.

Reason proposed that any or all of these layers could in practice have weaknesses of some kind. In general terms, “latent conditions” are weaknesses that affect the first three layers (for example, flawed decisions by managers) and “active failures” are those affecting work activities (for example, unsafe acts by workers). Defenses, meanwhile, can be affected by either type of weakness. The effect of latent conditions and active failures is to render the work system vulnerable to hazards; the more hazards are allowed into the system, and the further they are allowed to promulgate through it, the more likely that an accident will ultimately occur. Hence, organizational safety can be seen as a matter of ensuring that the components that are present within the organization (and, more specifically, those that are implicated in particular tasks) are made as robust as possible. The aim of this study was to examine the activities that take place in community pharmacy dispensing, and to identify the ways in which organizational components either contribute to or reduce safety.

## Methods

### Methodological framework

Quantitative studies tend to focus on objective abstractions such as metrics and scores that do not always capture the nuances embedded in daily practices.^17^ While such studies provide generalized information than can be translated into protocols, they sometimes overlook shifting or dynamic human factors that are needed to understand subtle processes in daily practices. To understand how different components interact in dispensing and identify risk issues implicated in them, this study adopted the qualitative approach of socio-technical or ‘socio-material’ paradigms. Socio-material paradigms are used to study networks of people and their relationships with other components in an environment.^18–21^ A common theme in these paradigms is their focus on studying both tangible (e.g. materials) and intangible (e.g. relationship) components of systems; such as components that enable people to adopt and embed certain practices into their daily routines. The application of such paradigms in research follows qualitative multi-method approaches. In pharmacy specifically, these paradigms have been used to characterize work practice in terms of safety cultures from human and organizational perspectives.^6,22^ Various studies demonstrate how the study of work practice can be approached in regard to data collection and analysis.^23,24^ Harrison et al, for example, used this approach to study networks of people and their attitudes, organizational factors affecting them and environments within which they operate in health care information technology systems. Using this approach Harrison showed how it was necessary to understand key interactions among the components such as the people who use the systems, their workflows, culture, social interactions, and technologies when studying health care IT systems.^23^ However, since these paradigms do not specifically focus on risk, the study also drew on concepts of risk contributors in and outside of clinical practice.^11,14,25–27^ From this background literature, the study team identified the key topics for examination in the study as people and their attitudes, physical environments, management structure and ethos, workload and workflow, technical systems; and, the processes and interactions that bind them in practice.

### Data collection

The study protocol was submitted to the Cambridgeshire NHS Research Ethics Committee (REF 08/H0304158) and was classed as a service evaluation. NHS Research and Development approvals were obtained prior to conducting data collection at the various study sites. The research described in the current paper was part of a larger study to investigate the impact of the national Electronic Prescription Service Release Two (EPS2) on general practices, community pharmacies, patients and business stakeholders.^28^ The sites sampled were all located in the Midlands and Northern regions of England. Six were independent pharmacies, and nine were chains consisting of local and national chains. Descriptive details of the pharmacies are published elsewhere.^29^ Data were collected from December 2009 to September 2011. Pre-EPS2 implementation data were collected from 15 pharmacies (2009–11), and post-EPS2 implementation data were collected from eight of these 15 pharmacies (2011). Seven of the pre-implementation sites did not go on to adopt EPS2 at the time of the study. The study was designed by a team of sociologists, patient safety and pharmacy practice experts. Data were primarily collected by JH, who is an experienced qualitative social scientist with health research experience. JH was helped on occasions by other researchers within the team, including SS (pharmacist), JD (pharmacist and doctoral candidate), RH (e-health expert), and JW (sociologist) with informal observations from MB (practicing and academic community pharmacist). Ethnographically-informed methods – non-participant observations, shadowing, and in-depth interviews – were used to collect data.

### Non-participant observations

Observations were used as an unobtrusive method to enable the observation of dispensing without interfering with the process. Key issues considered in the observation included:

• The physical environment.
• The management and their ethos toward innovation.
• The fluidity of the dispensing processes from when the prescription arrived in the pharmacy to when it was dispensed (bagged for collection or handed to the customer) for both acute and repeat prescriptions.
• The types and amount of dispensing conducted, hours of work, and types of services offered.
• The number of staff present at the time of observation, ranging from the counter assistant to the responsible pharmacist.
• The location and availability of material resources such as printers, computers, secured storage for controlled drugs, logging books for controlled drugs and pharmacy management software.
• How the electronic aspect of dispensing was engaged with, and the use of protocols and procedures while dispensing.
• The way staff interacted with each other and with patients/customers during dispensing.
• Attitudes toward risk and violations in context of all the above.

**Fig. 1:**
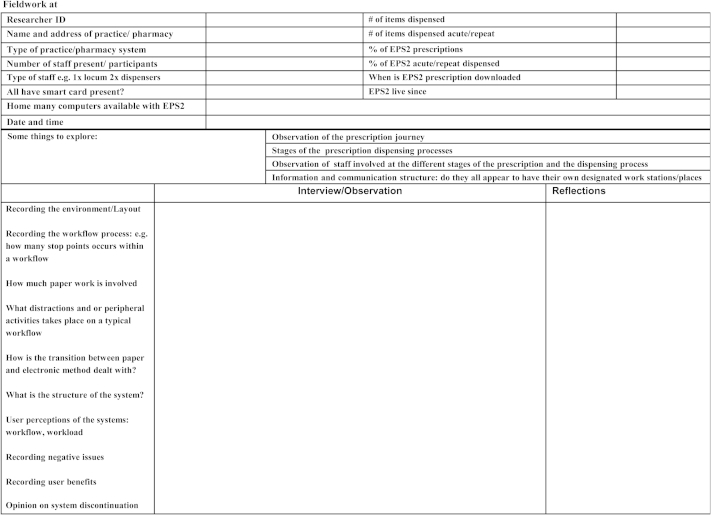
Fieldwork guide (template).

Observations were carried out using a fieldwork guide (Fig. 1), and consisted of two-three day visit to each site. All researchers reflected on the data collection process in a written form, after the data collection (Fig. 1).

### Shadowing

Shadowing is a technique used in social sciences and user experience research. It comprises the researcher following the user of a service or system as they use it. It is similar to a non-participant observation; however, unlike observations, the user is encouraged to speak their thoughts out loud while using the system: “Throughout the shadowing period the researcher asks questions which will prompt a running commentary from the person being shadowed.”^30^ Shadowing enables the researcher to see the system from the user's perspective. Due to the busy nature of pharmacies and the need for the researchers to position themselves to minimize interference to the clinical processes, the use of shadowing was limited. It was conducted during the beginning of the observation period when the researchers were being briefed on the pharmacy procedures at the beginning of the working day. For example, shadowing was used to record descriptive information about the pharmacies such as the types and amount of dispensing conducted before observing it in practice. It was also used to recorded field notes where the pharmacist or dispenser could not give an interview, but was able to talk in between dispensing tasks. Shadowing helped the researchers notice the subtle differences in the described formal procedures and adapted procedures in the routines of work practice. Data collected from this were analyzed together with observation data and informed the writing up the case studies.

### Interviews

Interviews were conducted to follow-up issues highlighted during shadowing and observation and to generally debrief pharmacists on data collection process. The interview questions were semi-structured and were based on themes in the fieldwork guide (Fig. 1), in addition to any other observations made. The responsible pharmacist in each pharmacy was interviewed in the pre- and post- data collection, and any dispensers that were available. Where the staff were too busy to give interviews, notes were taken and analyzed as short transcripts together with other field notes for each case study. In some cases, the responsible pharmacist was the same person in both the pre- and post-implementation interviews; in other cases, they were different people. Overall, 28 interviews were conducted in the pre-implementation study and 10 interviews in the post-implementation study. The interviews ranged from 10 to 45 minutes depending on how much time was available to the pharmacist or dispenser. All 38 interviews were radio recorded, and were transcribed verbatim.

### Analysis

Each observation was written into a case study averaging 3500 words. All case studies were co-written by the data collectors taking into account researchers' reflexivity. The case reports were then circulated to the wider team for comments in a draft form, and then finalized after receiving comments from members of the team. As part of project's protocol to provide formative feedback to implementer teams, findings were sent to NHS informatics leads for comments. Overall, 52500 words in the case studies were analyzed using cross-case thematic analysis based on the observation themes. A cross-case thematic analysis essentially analyzes commonalities and differences between cases studies, and within themes in the case studies.^31,32^ For this paper, additional analysis was conducted using a thematic framework approach by specifically looking at how latent conditions and active failures were implicated in the dispensing journeys in each case (and related field recordings). Interviews were analyzed using a combination of thematic and line-by-line analysis to draw out key themes. In addition to these, two presentations were conducted to debate and review the findings. The first presentation was given at the School of Pharmacy, University of Nottingham and had different types of pharmacist, patient safety and e-health innovation experts present. The second presentation was given at the School of Pharmacy, University of Manchester included group of pharmacists and patient safety researchers. Findings from all three methods were cross-validated with each other and synthesized to identify themes that had safety implications running through them.

## Results

From the 15 case studies, all the community pharmacies used Patient Medication Record (PMR) systems to dispense, which are provided by commercial software vendors. Different vendors supply their own PMRs. Pharmacists in the 15 case studies used a variety of PMR systems to dispense under different services such as ‘wait-in’ and ‘call-backs’ (described in Table 1). During dispensing, these different services were prioritized using arrangements like color baskets, and queuing items on counter tops. In one pharmacy, a robot was used. The average number of items dispensed per month ranged from 2000 to 26000 (the most items being dispensed in a robot-assisted pharmacy).

**Fig. 2:**
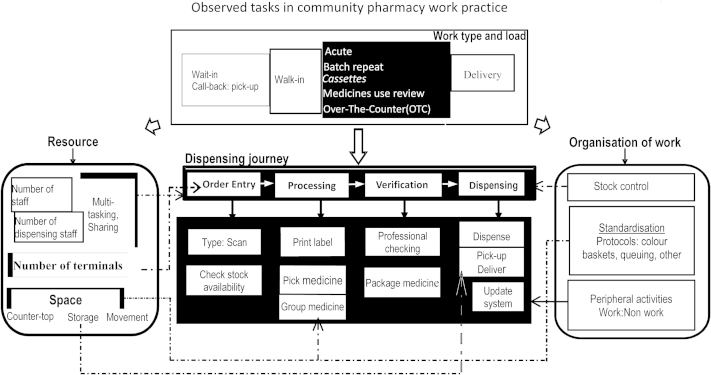
Observed system of dispensing in community pharmacies.

Overall, findings showed dispensing as a complex practice that relied on key components such as varied teams of people, dispensing processes, workloads, dispensing protocols, pharmacy resources, organizational systems, technologies and physical space to dispense a prescription (Fig. 2). These components are inextricably linked and as a result, it was difficult to identify whether a potential source of risk was a result of people's unsafe acts or from organizational (latent) conditions. What was clear, however, was that when relationships between the people component and other components were cohesive, steps involved in prescription dispensing journeys were clear and were operated to a high standard as this typical dispensing journey in Case 05 shows, recorded by JH:

1. Counter Assistant (CA) receives prescription, and places it in its relevant color basket.
2. CA then calls out the prescription (e.g. “we have a wait-in,” or, “we have a call-back”) and then queues the basket for processing according to its priority. ‘Wait-ins’ are placed immediately next to the computer terminal for the attention of the pharmacist.
3. Dispenser picks up the basket with the prescription, and walks around to locate medicine and pick from shelf (if it is a popular medicine) or checks the computer to see if it is in stock before picking from shelf.
4. Dispenser places medicines in basket and queues (according to priority) for pharmacist to professionally check.
5. Pharmacist checks, labels, packages, and hands over to customer. Call-backs are usually prepared and queued, so that the pharmacist would check and dispense when the customer calls back.

Across the 15 case studies, dispensing journeys were clear, smooth and ordered in most cases. There were, however, instances where people did not engage well with the other components. This included people not organizing dispensing processes properly due to unclear communication protocols, people finding it hard to move around due to lack of physical space, people finding it hard to cope with workload and multitasking, people's lack of technical know-how on computer system malfunctions, people engaging in unsafe acts due to lack of a management structure, and people having to manage risk from external sources such as prescriber mistakes. Since the ‘teams of people’ were inextricably linked to each activity in the dispensing journey, any issue with the people component had a direct effect on all other components, and sometimes snowballed into a challenging dispensing process. Four broad categories that emerged, and which demonstrate the interdependency of unsafe acts and latent conditions, are:

1. **Dispensing with divided attention**: this includes *latent conditions* such as relaxed management enabling people to indulge in *unsafe acts* such as dividing their attention between social interaction and dispensing; *unsafe acts* such as how clutter in the pharmacy created a *difficult spatial condition* for physical movement, forcing staff's attention to be divided between dispensing tasks and be constantly mindful of where they are stepping.
2. **Dispensing under pressure**: through *latent conditions* such as heavy workload or through sharing equipment, people were pressured and did not interact effectively with the system which resulted in *unsafe acts* such as not taking time to check medications properly. Through *unsafe acts* such as lack of communication, staff did not always communicate when they temporarily altered procedures; this generated *challenging latent condition* such as dispensing with confused procedures which put staff under pressure to keep on top of tasks.
3. **Dispensing in restricted spaces**: *unsafe acts* such not clearing away clutter in pharmacy or through *latent conditions* such as small physical dispensing space, were challenging to how people related to the restricted spaces while dispensing in terms of space for organizing work, physical movement and storing medicines.
4. **External influences**: *unsafe acts* from external sources such as general practices could potentially exacerbate errors within the pharmacy as errors from external sources might not be part of the pharmacy's (*organization's*) safety practice and so could be overlooked.

In the following sections, examples are given on some of the safety implications identified citing instances of people's unsafe acts and organizational conditions, using observation case studies, field recordings, and interview excerpts. It should be noted that not all themes in the data collection identified a potential risk, hence only the themes that showed risk issues and other ‘emergent’ themes from the analysis are discussed in the article.

### People and their approaches to work

Responsibilities for dispensing prescriptions were varied and depended on the environment. The number of staff in the pharmacy influenced the distribution of tasks. Different pharmacies had different workloads. Staff dispensed under pressure when there was not sufficient and appropriate number of staff for the workload. Walk-ins were especially demanding as the patient could not be left waiting in the pharmacy without immediate attention. From interviewing the pharmacist in case 01, it transpired that although around 30% are walk-ins and approximately 70% are deliveries, the walk-ins took precedence, and this upset the workflow. Also, the walk-ins took a large proportion of the dispensing time in relation to the deliveries, even though the latter were the biggest source of the dispensing income in that pharmacy. In general, pharmacy staff appeared rushed and harassed if they had a high number of walk-ins, particularly when they were also trying to deal with other types of dispensing such as bulk repeat collection and delivery, care home medications and MURs. Commonly, up to four people handled a prescription during its dispensing journey. There were, however, cases when the responsible pharmacist dispensed prescriptions alone and this meant that they were often dispensing under pressure and with divided attention. There was disruption in the dispensing journey when the pharmacist was solely in charge of dispensing, as often, the pharmacist was needed for other tasks. For example, case 08 is a supermarket pharmacy. There were three different temporary agency recruited pharmacists (called ‘locums’ in the UK) during the observation period, each locum running a shift on different days. On 19/07/2010, JH recorded:

10.58. Locum is left alone at pharmacy. Customer is at the counter, and so he goes to see to them. Phone is ringing but locum is busy. He has left the FP10 (prescription he is processing). The phone has stopped ringing just before he finished serving the customer. He goes back to dispensing the FP10. It is now 11.00.

11.00. A customer is at the counter. Locum stops dispensing to see to customer. Customer hands over FP10 but keeps chatting to the locum despite him [locum] showing his desperation to go. Customer now leaves (for a call-back). Another customer immediately walks in and asks for something. This has taken locum to a different area (looking through records). The dispensing still waits. 11.04. A new customer walks in with two FP10s. There are now a few customers waiting and only the locum. He is trying not to get stressed.

[Note to self: bring this issue up in the follow up interview].

11.08. Phone rings again. [Locum runs by me (JH) and says I can't split myself into two]. Locum now stressed.

11.09. Phone rings again. There are about five customers waiting. Problem is exacerbated by each customer wanting a chat and asking questions. Locum very patient and tries to give each customer all the time they demand.

11.15. There now three customers but other items on the FP10s are dispensed. Call-back lady at 11.00 now comes to collect. It is 11.15.

Dispenser now arrives from breakfast break. Work is now shared but there is now a queue of FP10s. Problem with medication as they may not have some in stock. Customer from 11.15 comes back at 11.35 only to be told that item (medicine) is not in stock.

In cases of multiple handlers, prescriptions were dispensed faster according to our timed recordings of when prescriptions arrived at the pharmacy to when they were dispensed. This, however, relied on clear communication protocols between the dispensing staff. Without clear protocols, the pharmacist sometimes would not prioritize an ‘urgent’ wait-in item for professional checking because they were unaware that the ‘urgent’ item was waiting as it had not been placed in the designated basket or location for “urgents.”

### Management structures

In terms of management and ethos of the pharmacies, within the immediate vicinity of the pharmacy, the pharmacist was responsible for overall dispensing decision-making. In instances where the responsible pharmacist could not be located or was occupied with a task such as MUR, dispensing halted until the pharmacist came back and then they sometimes rushed to perform final checks quickly to make up for lost time, resulting in checking medications under pressure. In terms of organizational and management structures, staff in chain pharmacies felt they had less autonomy and only had passive input to the organization's strategic goals concerning meeting operational targets such as delivery of MURs, recruiting patients' into a repeat management system, and maximizing monthly numbers of dispensed items. They felt that being on the constant lookout for events that might count toward targets set by the organization distracted their attention from immediate dispensing tasks. Case 09 shows an excerpt from interview conducted by JH on 19/07/2011. In this example, the pharmacist is talking about how meeting the 2011/12 annual maximum delivery of 300 MURs takes away concentration from immediate dispensing tasks such as patient care.

It is conflicting, because you care about your patients and you think it's not about targets. Like the old lady that came in and she was talking about her painkillers when she has a headache. I noted that down, I could use that as an MUR in the sense that I did sort of tell her that she's not supposed to be taking them all the time. It's good that you can use it, because it's exactly what it is, you are telling the patient how to use them and advising them about it. Those are the little things that I have to concentrate on and pick up on to meet my targets.

### The physical environment

The availability of physical space in which to carry out work influenced dispensing journeys in terms of safety. Space for physical movement, storage and processing medicines aided the fluidity of the dispensing process. In sites that had limited space, dispensing appeared chaotic as staff bumped into each other whilst going about tasks, or could not queue items on counter tops for processing and sometimes piled items into basket; the baskets were then piled on top of each other. Medicines were sometimes stored in cardboard boxes on the floor where shelves were full. Cases 03, 04, 08, and 13 were particularly lacking in space. In some cases, the physical space was restricted due to clutter and lack of organization within the pharmacy as in Case 03. In Case 04 however, the restricted space was due the small area designated for dispensing; JH recorded the following:

The small space means that sometimes medicines in baskets waiting to be verified are piled on top of each other or are placed very close together. One medicine could easily fall into another basket and if pharmacy manager is verifying the basket's contents with divided attention, this could be a source of risk. Leaving medicines in boxes on the floor due to lack of space does not provide a clear view of each medicine. One medicine could easily be mistaken for another. The storage of large number of dispensed medicines in boxes on the floor for the delivery driver could be another potential source of risk. These medicines are placed directly in an unmarked boxed and could easily be mistaken for new boxes of medicines (except for the labels on them).

### Engagement with technologies

Generally, staff felt that pharmacy technologies were useful and effective in managing dispensing. Sometimes, however, there were malfunctions in the computer systems, and pharmacists had to rely on the vendors to resolve these issues. In terms of safety, some pharmacists had to dispense without the means of checking prescriptions against patient records. For example, in case 07, JH recorded on 08/06/2010:

The system has been down for about 15 minutes and so a customer's prescription was dispensed without assistance from the computer. The pharmacist has been on the phone twice to complain and it is currently being seen to.

Sometimes staff had to share equipment which caused jostling between them. The unbalanced ratio of people to equipment put staff under pressure to carry out tasks quickly with little time for comprehensive safety checks. For example in case 05 JH recorded on 08/04/2010:

11.11. While staff uses computer, another staff drums her fingers on the counter waiting to use the terminal. She (first staff) jokes “No pressure” while a third staff waits.

### Attitudes toward safety

The attitudes and motivation of staff toward safety while dispensing appeared to vary. The pharmacies had designated protocols, but some staff were less strict in their application of these protocols than others. For example, nine of the sites used color baskets to prioritize dispensing. They did this in terms of urgency of walk-in prescriptions (e.g. red basket = wait-in, gray basket = call-back), or to denote the prescription's final destination for prescriptions that are dispensed in bulk and delivered to the patient (e.g. blue basket = Alpha's surgery, Yellow basket = Zulu's care home). Three pharmacies had no clear system and took to queuing prescriptions and items on the counter top. Safety implications were identified in cases where protocols were not followed and procedures violated. For example in case 06, JH recorded on 22/04/2010:

There are color baskets red, white and blue which are supposed to be used to organized dispensing, but I have just been informed that this procedure is not usually followed.

There were also procedure violations in relation to labeling. Labels were sometimes printed off long before the medicine was processed and dispensed. These labels were pinned onto the edge of counter tops or other surfaces until they were needed, thereby increasing the risk of labeling medicines with the wrong labels.

Social activities were sometimes incorporated into work such as personal telephone calls, conversations between staff about non-dispensing issues and other non-work-related activities such as going on the Internet to check personal emails. Some listened to the radio, and staff in one pharmacy watched television. Some social activities were used to project a family atmosphere to propel work and influenced how dispensing was approached. It must be noted that social activities and violations observed in the pharmacies are a snapshot of two-three day observations and may not be prevalent in the long-term or in all pharmacies. In case 06, for example, JH recorded on 26.04.2010:

Viewing television seems to be part of work culture. From about 9.15 to noon, the staff was viewing television whilst dispensing medicines. During the television viewing period, attention of the staff was very distracted. JH recorded: 9.18. There is a television playing on the dispensing counter. At a later time (time not recorded), JH added: There is constant attention on the TV. At one point a customer was left waiting as Counter Assistant (CA) was occupied watching TV. She (CA) has been reminded that a customer was waiting. The television was firmly stationed on the front counter and appeared to be part of the pharmacy's hardware. To confirm this, a researcher collecting data for a different work stream of this project reported watching television (during his lunch break) at this pharmacy.

It should be noted that in other areas this pharmacy also demonstrated a positive work practice. For example, the temporary pharmacist (locum) strictly followed procedures when dispensing controlled drugs by supervising the patient when they took the medication and then logging it on a report form before going back to dispense other medications.

### Other factors: Prescriber influences

Prescribers' instructions were sometimes not clear, or on occasions change their mind about a prescription, and required the pharmacist to intervene. This sometimes meant the pharmacist had to be on a special lookout for a specific patient in addition to their duties and worked with divided attention. In case 07, on 10/06/2010 JH recorded:

11.24: There has just been a phone call from GP who asked if a ‘Joe Bloggs’ has been in. After the call, the pharmacist informed me that the GP had prescribed acute antibiotic but had changed their mind after the patient had left. As the patient was local, the doctor called the pharmacy to ask if the medicine could be stopped when the patient comes in. The patient could however choose to go somewhere in which the medicine would not be stopped.

## Discussion

### Key message from the study

Since potential sources of risks identified were linked to people's relationships with other key components in the system, the findings suggest that the method by which people interact and engage with other components in dispensing is the key contributor to risk. Similar to other relevant studies, components identified as potential sources of risk included sources of distraction which might lead to unsafe acts such as action slips and memory lapses; violations, or latent (organizational) conditions such as heavy workloads, inadequate knowledge, a stressful environment, or inadequate systems of communication.^3,11,14^

The four categories that emerged from the analysis show that safety management must address various components that make up dispensing and the relationships that bind them in operation. Human beings in working environments cannot completely avoid making errors. However, unsafe acts or unfavorable latent conditions could be addressed through the creation of better relationships between key components in practice. Community pharmacies are complex places of work because the pharmacy is essentially a business providing health care services.^33^ However, although the community pharmacy is a health care service provider, the pharmacist is usually the only trained clinician as support staff are generally non-clinical unlike clinically trained general practice (GP) nurses or dental nurses. Nevertheless, dispensing involves complex team work of clinical processes such as differentiating between similar medications, processing the correct dosage and monitoring patients, all of which are delivered under complex arrangements such as wait-ins, call-backs, MURs and care home medications. Staff, therefore, need to relate well to other components that are involved in dispensing under these complex arrangements such as communication protocols, workload, workflow, physical environment, management and organizational structure. It has been found that when employees work with high workloads or under time pressure, their rate of error increases due to violations, cognitive lapses and the general stress.^8,34–38^ Additionally, the physical work environment can have a key role in safe practices.^39^ While the increasing adoption of advanced IT systems, and other safeguards appears to have shifted focus away from the role of people in safety systems, it is the people who have to operate the IT and the safeguards in the first place, and must therefore relate to them favorably. A key message from this study is that in a complex environment such as the community pharmacy, the relationships and interactions between human and other components should be taken into consideration when developing a safety system.

### Adopting research into practice

Whilst pharmacies may not be considered as ‘high risk’ environments compared to, for example, the aviation industry, they could benefit from adopting an approach where the networks of people, and how they relate with other components in dispensing, are focused on practice and policy. An example of a way these research findings can be adopted into practice and policy is to improve the relationships and interactions between key contributors to dispensing through the creation of ‘standard dynamic’ environments. Standard dynamic environments are environments with unique identities, but where key interactions and relationships are similar, thereby enabling shared values and seamless transitions from one pharmacy to the other without the cognitive challenge of learning a new safety system. Standard dynamic environments would be where there is a core ‘standard’ system of what constitutes best practice through the amalgamation of new and existing protocols such as Standard Operating Protocols (SOPs), pharmacy design guidelines and other standardized procedures and guidelines,^1,9,40^ and which people could make dynamic by adapting it to suit the changing environment. Pharmacies could then consider (and potentially justify) the extent to which they have deviated from the core standard system as part of their safety practice for industry regulators.

### Strengths and limitations of the study

Using observations to study community pharmacy work practice allowed researchers to obtain perspectives on safety practices that exist within community pharmacies that are difficult to obtain with other methods; when people are asked to describe work practice in surveys interviews or focus groups, they often leave out the small ‘irrelevant’ routines which are often key to understanding work practices. Findings from this study could be used to inform the design of interventions aimed at improving safety practices in community pharmacies such as creating standard dynamic systems. The study was qualitative, which limits the generalizability of the findings. The results provide only a snapshot of community pharmacy safety issues, some of which might not be widely prevalent. It must also be noted that the data for this study was a result of an ethnographically-informed e-health study, therefore, it is possible that other key factors were overlooked during the data collection.

## Conclusion

Dispensing journeys of prescriptions rely on teams of people, processes, workloads, protocols, pharmacy resources, organizational systems, technical systems and space. Through this complex practice, it was difficult to ascertain whether sources of risk were down to peoples' unsafe acts or unfavorable working conditions as both sources were inextricably linked. What was clear, however, was how the relationships between the teams of people and other components are important in dispensing safely. This is because risk was identified when people did not interact well with other components, which meant that they dispensed prescriptions with *divided attention*, *under pressure*, in *restricted spaces* and having to *manage risk from external sources* such as prescriber mistakes. In conclusion, it is suggested that pharmacies reflect on the organization of their systems and review in particular how they can improve on the four key categories identified in this study.

## Figures and Tables

**Table 1:** Examples of services offered by community pharmacies

Examples of type of dispensing service offered	Description
Walk-in	Customers who walk in into the pharmacy to use it services such as buy Over The Counter (OTC) medicine, hand in a prescription, or pick-up a dispensed item. Walk-in customers fell into two categories, those who preferred to wait-in for their medicines to be dispensed, and those who preferred to hand in their prescription and call-back to pick-up the dispensed medicines later. A call-back may take anything from 15 min from after the prescription is received by the pharmacy to a few days, or until the customer comes to collect. For the purposes of this paper: a wait-in is used to describe those who wait-in the pharmacy for the prescription to be dispensed, a call-back denotes those who return after 15 min (or the same day) to collect after running other errands in the area, and pick-ups denotes those who collect their dispensed prescriptions after one day. A walk-in may also be delivered to the customer after dispensing.
Batch/Bulk repeat	Denotes repeat prescriptions collected from general practices in batches and dispensed. After dispensing, some of these were stored for the customer to pick up. Others were delivered to the customer. Some were urgent and needed to be dispensed and delivered the same day.
Care home medications (or cassettes)	Are containers that separate the medicines into different compartments depending on when they are to be taken (day/time); they are generated from repeat prescriptions for customers who might have problems taking the correct dosage, and were therefore processed and dispensed separately.
Medicines Use Review (MUR)	A reimbursed service, were conducted by the pharmacist and included checking patient satisfaction with a drug they have been repeatedly prescribed after a long duration. MUR is useful for those on repeat prescription managed by the pharmacy as the patient does not see their physician regularly.
